# Case report and literature review: patient with gastroduodenal intussusception due to the gastrointestinal stromal tumor of the lesser curvature of the gastric body

**DOI:** 10.1186/s12893-019-0608-3

**Published:** 2019-10-29

**Authors:** Mihajlo Đokić, Jerica Novak, Miha Petrič, Branislava Ranković, Miha Štabuc, Blaž Trotovšek

**Affiliations:** 1 Department of Abdominal Surgery, Ljubljana University Medical Center, Zaloška cesta 7, 1000 Ljubljana, Slovenia; 2 Department of Surgical Oncology, Ljubljana Institute of OncologyActa Chir Belg, Zaloška cesta 2, 1000 Ljubljana, Slovenia; 3 Institute of Pathology, Faculty of Medicine, University of Ljubljana, Korytkova 2, 1000 Ljubljana, Slovenia; 4 Department of Radiology, Ljubljana University Medical Center, Zaloška cesta 7, 1000 Ljubljana, Slovenia

**Keywords:** Gastroduodenal intussusception, Gastric gastrointestinal stromal tumor, Gastric outlet obstruction

## Abstract

**Background:**

Intussusception in adult patient is rare. Gastroduodenal intussusception due to the gastrointestinal stromal tumors is infrequently described in the literature. Authors present a case of gastroduodenal intussusception due to the low-risk gastrointestinal stromal tumor of the lesser curvature of the gastric body with literature review.

**Case presentation:**

Sixty-two-year-old male was admitted to our hospital with symptoms of acute gastric outlet obstruction. Imaging studies confirmed a lesion of the gastric wall producing gastroduodenal intussusception with pylorus obstruction. Upon laparotomy a tumor mass of the lesser curvature of the gastric body that invaginated through the pylorus into the duodenum was found. Desinvagination and resection of the tumor with the adequate resection margins were performed. Histology reveled a low-risk gastrointestinal stromal tumor. Due to favorable outcome only observation was suggested by the multidisciplinary team.

**Conclusions:**

Gastroduodenal intussusception due to the gastrointestinal stromal tumor of the gastric wall is a rare event. Surgical resection is the treatment of choice. In selected cases laparosopic resection of the tumor can be performed.

## Background

Intussusception rarely occurs among the adult patients. It accounts for 5% of all intussusception cases and in only 1% causes intestinal obstruction [1]. Gastroduodenal intussusception is the most infrequent form of intussusception in adults, it occurs in less than 10% [2]. Clinical and radiological findings in a patient with gastric outlet obstruction, gastroduodenal intussusception and gastrointestinal stromal tumor (GIST) of the lesser curvature of the gastric body is presented.

## Case presentation

62-year-old Caucasian male presented to the emergency room with acutely worsening epigastric pain lasting for several days and black stool lasting for a week. Symptoms of vomiting, inappetence and weight loss that have been lasting for the past six months without doctor appointment was also reported in medical history. Patient had a history of diabetes mellitus on insulin therapy. Upon clinical examination abdomen was not distended, there was no signs of guarding or rebound tenderness. Laboratory data showed anemia (hemoglobin 119 g/L, normal range 130–170 g/L; hematocrit 0.343, normal range 0.4–0.5), leukocytosis (13.5 10^9^/L, normal range 4.0–10.0) and normal value of C-reactive protein (below 5 mg/L, normal range 0–5 mg/L). Tumor markers CEA and Ca 19–9 were within normal range.

**Fig. 1:**
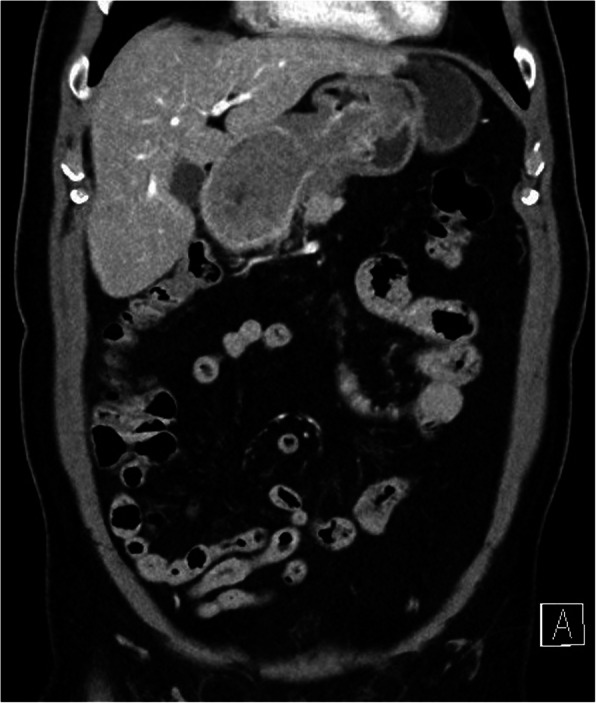
A CT scan demonstrating an intraluminal tumor of the lesser curvature of the gastric body producing a gastroduodenal intussusception with gastric outlet obstruction

**Fig. 2:**
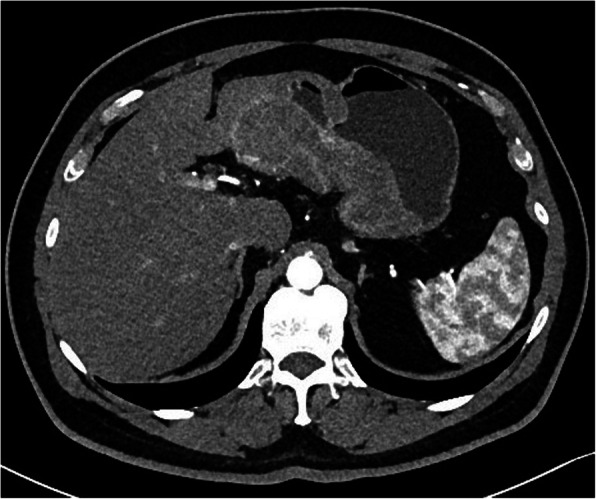
A CT scan demonstrating an intraluminal tumor of the lesser curvature of the gastric body producing a gastroduodenal intussusception with gastric outlet obstruction

Due to melena lasting for a week, patient underwent esophagogastroduodenoscopy (EGD) and ultrasound of the abdomen on the outpatient bases only few days prior to admission to the hospital. EGD was technically demanding due to the poor passage of the endoscope through the stomach, duodenal bulbous and upper part of the duodenum. Inflation of the gastric body was not possible, therefore the visualization of the gastric wall was poor with no obvious intraluminal mass or hemorrhage detected. Additionally gastric peristalsis was described to be absent. Abdominal ultrasound showed tumor formation of the gastric body, measuring 7 × 5 cm, but no intussusception was described. CT scan revealed a 5.4 × 5.6 × 6.2 cm intraluminal tumor formation of the lesser curvature of the gastric body with well defined borders was described. Tumor mass caused invagination of the gastric cardia through the antrum and pylorus into the D2 part of the duodenum producing gastric outlet obstruction (Figs. 1, 2). No dissemination to the parenchymal organs was described.

**Fig. 3:**
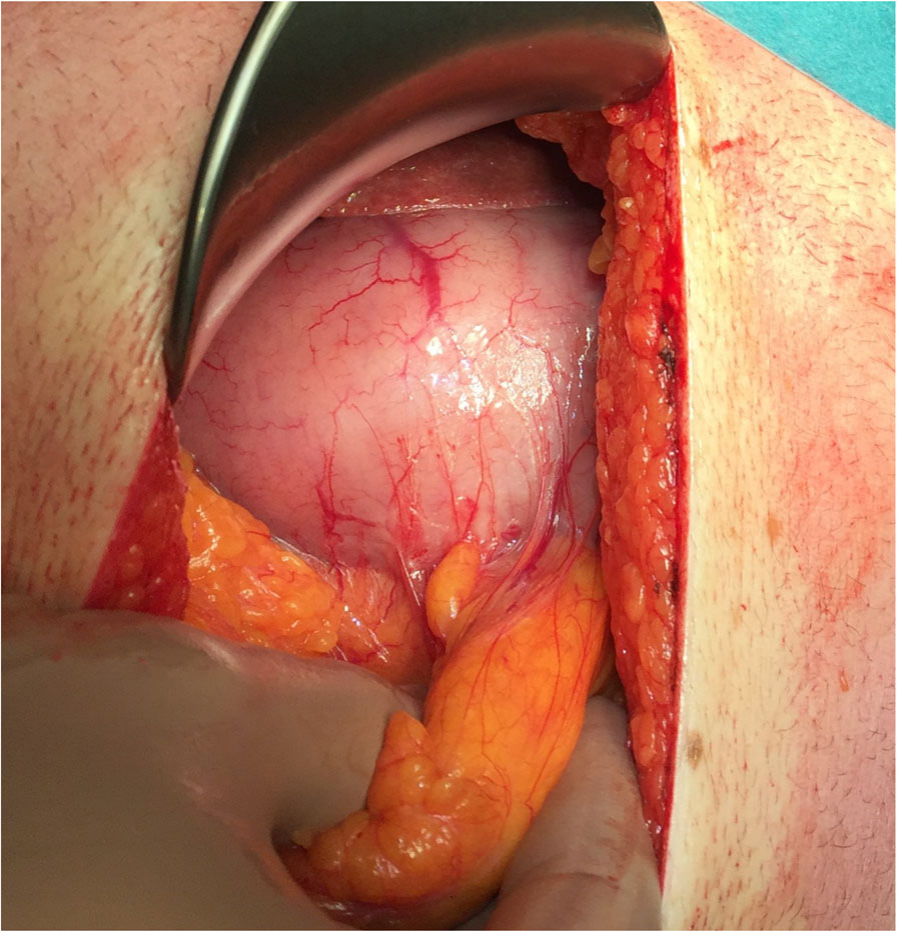
Palpable intraluminal gastric tumor with the impaction of mass through the pylorus into the duodenum with no other pathological finding in the abdominal cavity

**Fig. 4:**
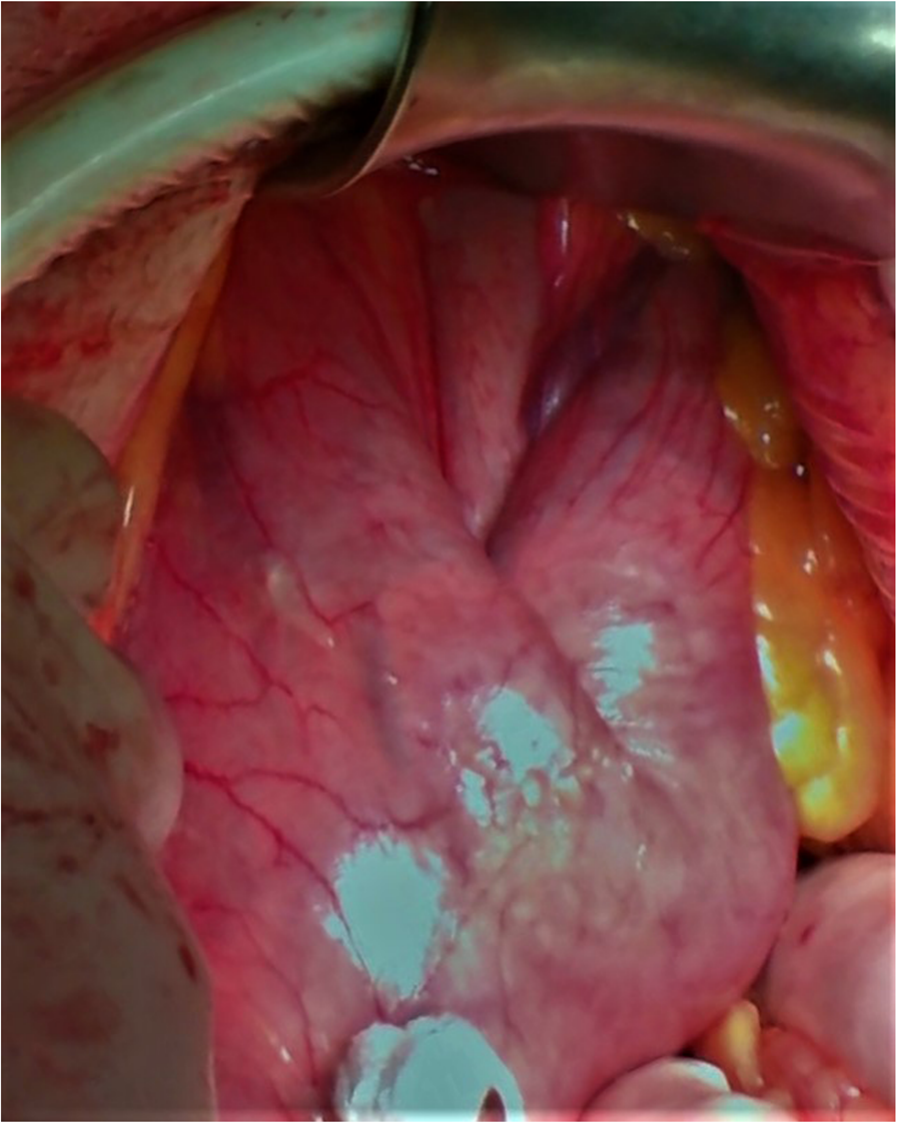
Palpable intraluminal gastric tumor with the impaction of mass through the pylorus into the duodenum with no other pathological finding in the abdominal cavity

Explorative laparotomy was performed. Palpable gastric mass with impaction of the tumor through the pylorus into the duodenum without signs of disseminated disease in the abdomen. Was found (Figs. 3, 4). Kocher mobilization of the duodenum and the head of pancreas was necessary for the successful desinvagination of the tumor. Anterior gastrotomy reveled a solid, well defined, intraluminal tumor of the lesser curvature of the gastric body just below the gastroesophageal junction. Circular radical resection of the tumor with one centimeter resection margin was performed. The gastric wall defect was sutured in transverse manner in two layers. Postoperative period was uneventful and patient was discharged on the ninth postoperative day.

**Fig. 5:**
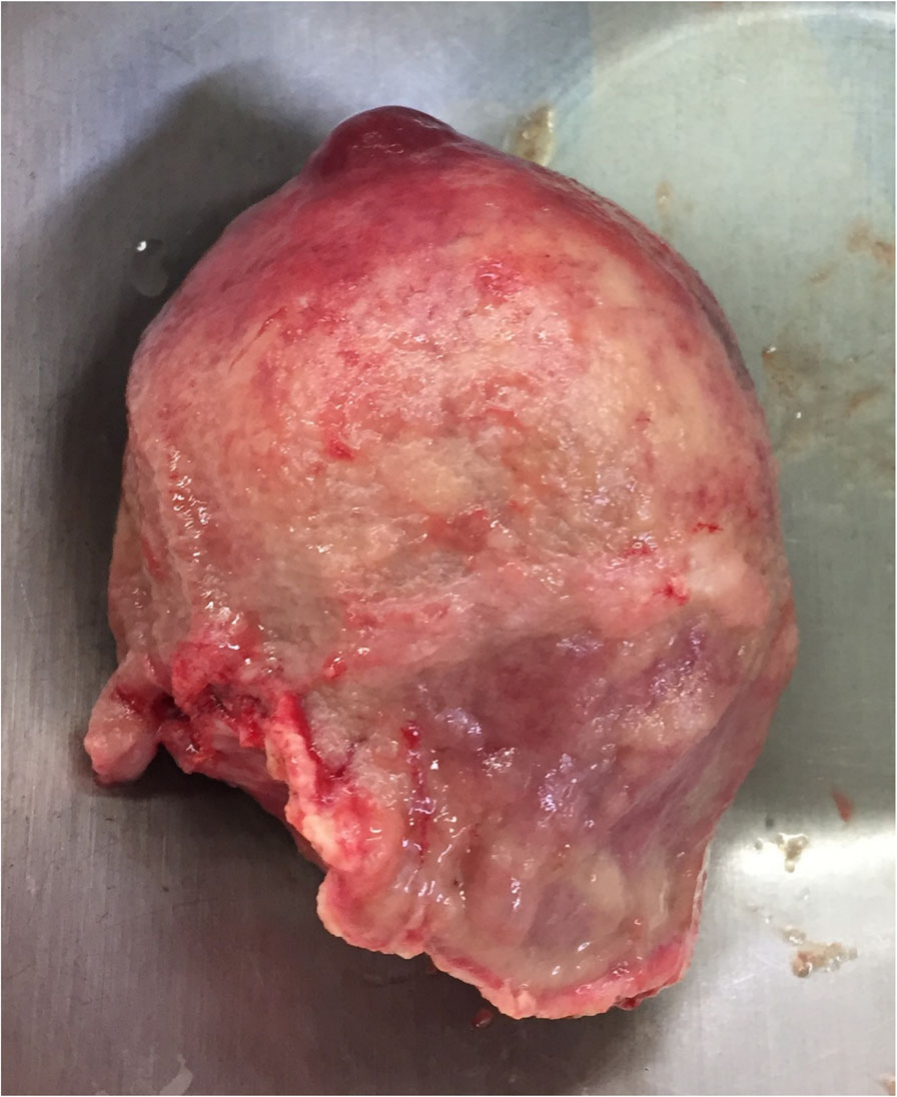
Solid mass with well defined borders

**Fig. 6:**
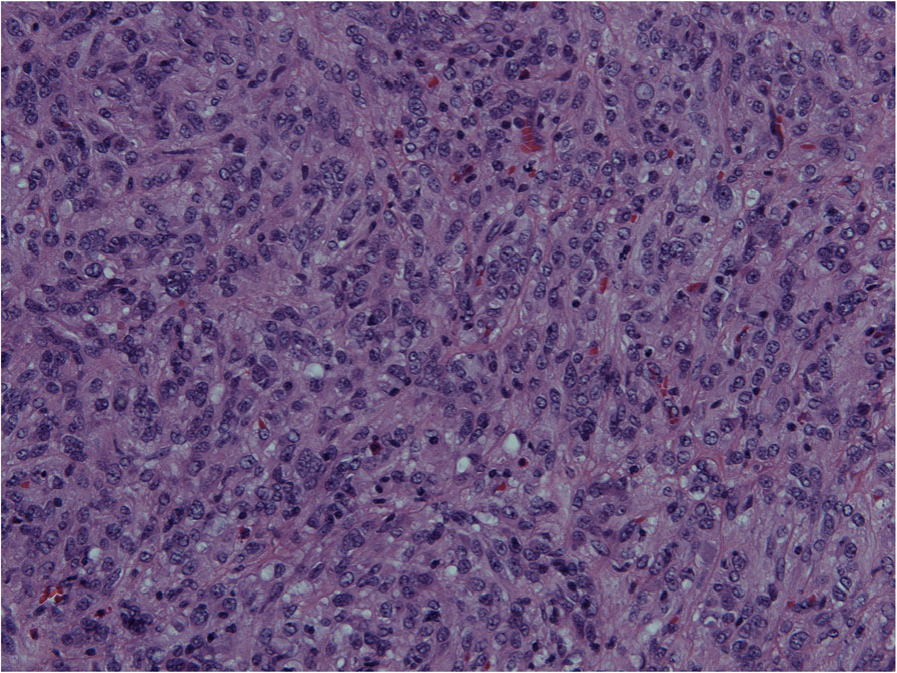
Microscopic image of GIST, HE staining, × 20 magnification

**Fig. 7:**
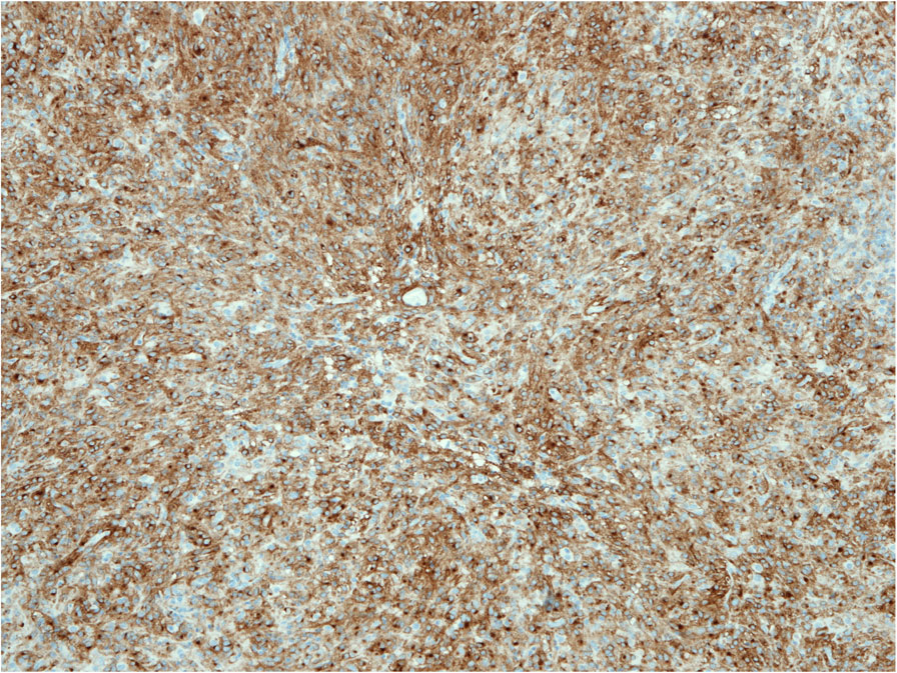
Microscopic image of GIST, imunohistochemical staining, CD117 positive, × 10 magnification

**Fig. 8:**
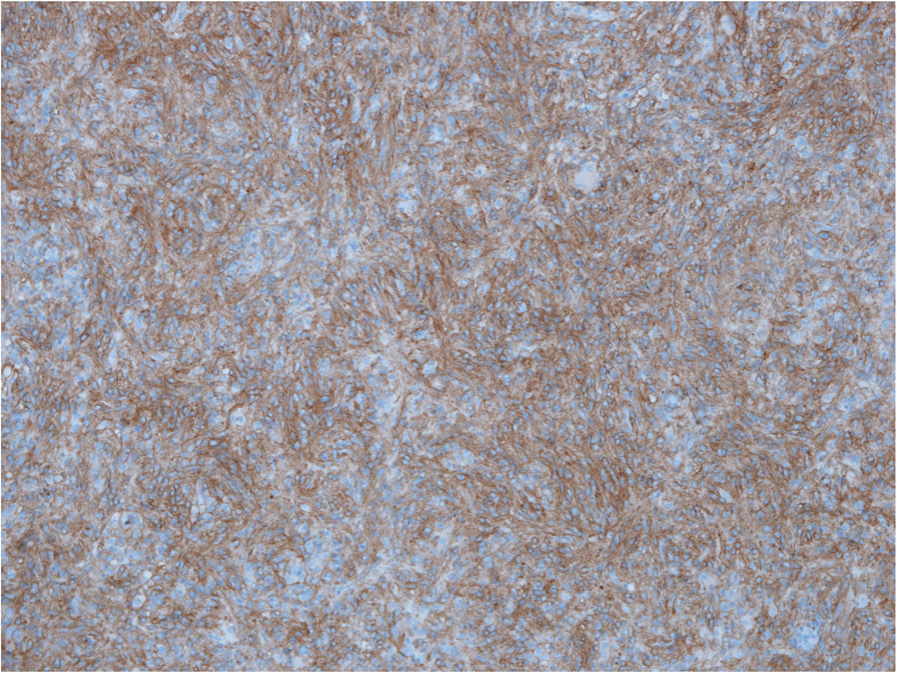
Microscopic image of GIST, imunohistochemical staining, DOG1 positive, × 10 magnification

Sample was send for pathohistological examination. Macroscopically specimen contained a well-defined 7.5 × 5.5 × 4 cm solid, grey mass with no necrosis (Fig. 5). Microscopical examination with hematoxylin and eosin staining (HE) and imunohistochemical staining revealed GIST with expression of CD117 and DOG1 (Figs. 6, 7 and 8). Tumor invaded the submucosal layer and muscularis propria but no necrosis or lymphovascular invasion was observed. Mitotic rate was 0/5/mm^2^. Proliferation marker Ki67 was less than 5%. Resection margins were free of disease. Tumor was pathologic staged as T3.

Patient case was presented on the multidisciplinary team meeting and due to low-risk GIST with favorable prognosis only observation is suggested.

## Discussion and conclusions

Authors report a case report of 62 years old patient with gastric outlet obstruction due to the gastroduodenal intussusception caused by a large, low risk GIST of the lesser curvature of the gastric body.

Intussusception in adults is an infrequent condition, therefore when the patient presents with the symptoms of the upper gastrointestinal obstruction, other causes should be considered [3]. Gastrointestinal intussusception is a very rare find where malignant or benign lesion of the gastric wall initiates the prolaps of the gastric wall through the pylorous into the duodenum. According to the literature, gastroduodenal intussusception accounts for only 10% of documented intussusceptions in the adults [2]. GIST of the gastrointestinal tract are rare, reported incidence is highest in Asia [4]. Most GIST arise from gastric wall (56%), followed by the small intestine and colon [4]. By authors best knowledge, GIST was described, as a leading point for gastroduodenal intussusception in only 17 cases (Table 1) [5–21].

**Table 1:** Review of the case reports on gastroduodenal intussusception with GIST

Reference	Age (years)	Sex	Location	Size (cm)	Presentation	Treatment
Yildiz [5]	85	F	Fundus	6 × 5	Symptoms of acute pancreatitis, weight loss for 6 months	Subtotal gastrectomy
Rittenhouse [6]	52	F	Fundus	5 × 5	Epigastric pain and vomiting for 1 day	Laparoscopic wedge resection
Crowther [7]	59	F	Anterior wall of antrum	6	Intermittent epigastric pain with vomiting for 3 weeks	Partial gastrectomy
M S [8]	74	M	Posterior wall	No data	Intermitent vomiting for 3 weeks	Partial gastrectomy
Chan [9]	34	F	Posterior wall of fundus	6.5 × 4.4 × 3.8	Epigastric pain	Laparoscopic wedge resection
Basir [10]	62	F	Posterior wall of distal body	5.2 × 3.5 × 3.2	Epigastric pain with melena for 3 days	Bilroth’s II partial gastrectomy
Adjepong [11]	84	M	Antrum	4x3x3	Intermitent abdominal pain, vomiting, weight loss and melena for 6 weeks	Laparoscopic bilroth’s II partial gastrectomy
Wilson [12]	78	F	Antrum	4.4 × 3.3 × 3.4	Epigastric pain and vomiting for 1 week	Laparoscopic wedge resection
Yamauchi [13]	95	F	Posterior wall of distal body	4.2 × 3.9	Vomiting, loss of apetite and melena for 1 week	Endoscopic submucosal dissection
Gyedu [14]	59	F	Anterior wall	7x6x5	Intermitent vomitig for 5 months	Partial gastrectomy
Siam [15]	29	M	Antrum	6 × 6	Intermittent epigastric pain, vomiting and melena for 5 months	Bilroth’s I partial gastrectomy
Zhou [16]	69	M	Posterior wall of antrum	4.5 × 4	Acute abdominal pain with vomiting for 6 h	Wedge resection
Jameel [17]	65	F	Anterior wall of antrum	6x6x4	Epigastric pain and intermittent postprandial vomiting for 6 months	Wedge resection
Shum [18]	34	F	Fundus	5 × 5	Intermittent epigastric pain	Partial gastrectomy
Ssentongo [19]	85	F	Fundus	2.5 × 2.5	Epigastric pain and melena for 1 day, postprandial vomiting for 14 days	Wedge resection
Komatsubara [20]	90	F	Fundus	5 × 4.5 × 4	Vomiting, loss of appetite	Wedge resection
De U [21]	42	F	Anterior wall of antrum	8x7x4	Abdominal pain for 6 months	Wedge resection

Reviewing the literature, patients with GIST and gastroduodenal intussusception most commonly presented with nonspecific symptoms of acute or intermittent abdominal pain with vomiting lasting from a day to several months (Table 1) [5–21]. Similar to our patient in some reports GI bleeding was noticed (Table 1) [9, 10, 13]. In the published cases of GIST producing gastroduodenal intussusception reported median age was 65 years (mean 64.5 years, range 29–95 years), predominantly female (76.5%) [5–21].

Standard histopathological examination of the specimen and H&E staining are not enough for the diagnosis of GIST. Immunohistochemical studies are the golden standard [22]. GIST is typically confirmed by positive staining for CD117 antibody as was the case in this report [23]. As proposed in the literature, 4–15% of GIST can be CD117 negative, therefore additional immunohistochemical staining for DOG1 antibodies should be performed to confirm the diagnosis [24].

In case of gastroduodenal intususception, reduction of the invagination can be performed endoscopically. In described case, where a large GIST led to the intussusception and protrusion of the gastroesophageal junction through the pylorus, endoscopic reduction of the invagination was not feasible. Moreover, upon endoscopy, the stomach was not visible and the tumor was not recognized. Both imaging techniques, ultrasound and CT scan of the abdomen, respectively, described tumor formation of the gastric wall with CT scan describing the gastroduodenal intusussception. Desinvagination and desimpaction of the tumor was not possible until Kocher mobilisation of the duodenum and pancreatic head was performed.

Surgery remains the treatment of choice for resectable GIST with no dissemination but also for oligometastatic disease with potentially resectable liver metastases. In many cases, especially in low-risk GIST, surgery represents the definitive treatment. A wide local resection with clear resection margins is recommended. In cases of invasion to the adjacent organs en-block resection should be performed. Systemic therapy may be indicated in patients with high-risk tumors and in patient with unresectable tumor or metastatic disease. Small lesions can be resected laparoscopically. Endoscopic resection of the GIST is not recommended [25]. In the literature only one case of endoscopic resection of the GIST producing intussusception was described. Endoscopic resection was performed in case of a 95-year-old patient who declined surgery. Tumor mesured 4 cm and was successfully reduced endoscopically. Due to the repeated invagination and surgery refusal only submucosal dissection of the lesion for the purpose of local treatment was performed [13]. Upon the literature review in cases of GIST producing gastroduodenal intussusception, lesions measured more than 5 cm (median 5.1 cm, mean 5.3 cm, range 2.5–8 cm) [5–21]. As in described case, in majority of patients with large lesions, exploratory laparotomy was performed. The extent of resection ranged from wedge resection to subtotal gastrectomy [5–8, 10, 14–21]. In one third of described cases (29.5%), laparoscopic resection was performed (Table 1).

GIST of the gastric wall rarely produces gastroduodenal intussusception. As shown in our case patients with low-grade GIST have favorable prognosis and in most cases radical surgical resection is the only treatment needed. When GIST arise from the gastric wall, resection of the tumor with adequate resection margins should be performed. For small lesions where desinvagination can be achieved endoscopically, laparoscopic resection of the tumor can be considered. In cases of large, impacted lesions, open approach with desinvagination and tumor resection is advisable.

## Data Availability

All available data are presented in the case.

## Abbrevations

EGD: Esophagogastroduodenoscopy
GIST: Gastrointestinal stromal tumor
HE: Hematoxylin and eosin staining
